# Identification of High Tolerance to Jujube Witches’ Broom in Indian Jujube (*Ziziphus mauritiana* Lam.) and Mining Differentially Expressed Genes Related to the Tolerance through Transcriptome Analysis

**DOI:** 10.3390/plants12112082

**Published:** 2023-05-24

**Authors:** Yaru Xu, Chao Wang, Decang Kong, Ming Cao, Qiong Zhang, Muhammad Tahir, Ying Yang, Shuang Yang, Wenhao Bo, Xiaoming Pang

**Affiliations:** 1 State Key Laboratory of Tree Genetics and Breeding, National Engineering Research Center of Tree Breeding and Ecological Restoration, Key Laboratory of Genetics and Breeding in Forest Trees and Ornamental Plants, Ministry of Education, College of Biological Sciences and Biotechnology, Beijing Forestry University, Beijing 100083, China; 17862824095@163.com (Y.X.);; 2 National Foundation for Improved Cultivar of Chinese Jujube, Cangzhou 061000, China; 3 Shandong Institute of Pomology, Taian 271000, China

**Keywords:** jujube witches’ broom, high-tolerant cultivar, phytoplasma concentrations, key genes

## Abstract

The jujube witches’ broom (JWB) disease is a severe threat to jujube trees, with only a few cultivars being genuinely tolerant or resistant to phytoplasma. The defense mechanism of jujube trees against phytoplasma is still unclear. In this study, we aimed to investigate the tolerance mechanism of Indian jujube ‘Cuimi’ to JWB and identify the key genes that contribute to JWB high tolerance. Based on the symptoms and phytoplasma concentrations after infection, we confirmed the high tolerance of ‘Cuimi’ to JWB. Comparative transcriptome analysis was subsequently performed between ‘Cuimi’ and ‘Huping’, a susceptible cultivar of Chinese jujube. Unique gene ontology (GO) terms were identified in ‘Cuimi’, such as protein ubiquitination, cell wall biogenesis, cell surface receptor signaling pathway, oxylipin biosynthetic process, and transcription factor activity. These terms may relate to the normal development and growth of ‘Cuimi’ under phytoplasma infection. We identified 194 differential expressed genes related to JWB high tolerance, involved in various processes, such as reactive oxygen species (ROS), Ca^2+^ sensors, protein kinases, transcription factors (TFs), lignin, and hormones. Calmodulin-like (CML) genes were significantly down-regulated in infected ‘Cuimi’. We speculated that the CML gene may act as a negative regulatory factor related to JWB high tolerance. Additionally, the cinnamoyl-CoA reductase-like SNL6 gene was significantly up-regulated in infected ‘Cuimi’, which may cause lignin deposition, limit the growth of phytoplasma, and mediate immune response of ‘Cuimi’ to phytoplasma. Overall, this study provides insights into the contribution of key genes to the high tolerance of JWB in Indian jujube ‘Cuimi’.

## 1. Introduction

The jujube witches’ broom (JWB) disease, caused by ‘Candidatus Phytoplasma ziziphi’, is very harmful to Chinese jujube (*Ziziphus jujuba* Mill.) trees [1]. After phytoplasma infection, jujube trees exhibit various symptoms, such as yellowing, bunching, and variegated flowering leaves [2,3]. Young trees may die within 1–2 years after infection, while the growth of large trees is affected within 5–6 years, resulting in significant losses to the jujube industry. Currently, except for Xinjiang, the entire jujube industry in China faces a serious threat of JWB infection. The JWB phytoplasma is phloem-limited and is spread by a specific leafhopper, *Hishimonus sellatus* [1]. Moreover, in vitro cultivation of phytoplasma has been shown to be particularly challenging, which has impeded the development of effective control strategies, making it difficult to fully control the spread of phytoplasma [4,5]. Therefore, the identification of resistant germplasm is crucial for sustainable control and management of phytoplasma-associated diseases.

To date, no Chinese jujube cultivars have been reported to be symptomless or uninfected following phytoplasma exposure. Zhao et al. [6] and Wang et al. [7] documented the discovery of resistant cultivars that initially exhibited symptoms but later recovered to normal growth. Wild and related species are valuable sources of resistance genes, such as the Huanglongbing-resistant germplasm identified in citrus relatives [8]. When Chinese paliurus (*Paliurus hemsleyanus* Rehd.) was used as a rootstock, the Chinese jujube scions showed tolerance to JWB, even though the scions were susceptible [9]. Chinese jujube and Indian jujube (*Ziziphus mauritiana* Lam.) belong to the same genus. Chinese jujube, native to China with a long history of cultivation, has fruits that are high in nutritional content and has ecological, economic, and medicinal values [10,11]. Indian jujube is a small evergreen tree that is indigenous to India and has tropical and subtropical origins [12]. Jamadar et al. [13] reported the infection of jujube witches’ broom phytoplasma in Ber (*Z. mauritiana* Lam.) in India, but JWB infection in *Z. mauritiana* Lam. has not yet been reported in China.

In plant-phytoplasma interactions, plants have evolved various defense mechanisms to provide immunity against phytoplasmas infection, such as calcium (Ca^2+^) inward flow, reactive oxygen species (ROS), and secondary metabolite or hormone signaling pathway synthesis [14]. Pathogen-infected plants can trigger high ROS production, which directly harms bacteria [15,16]. Previous studies have found that disease/defense genes, plant hormone genes, and photosynthetic responsive and metabolism genes such as thioredoxin, beta-amylase, auxin, and abscisic acid may be involved in the interaction between jujube and phytoplasma [17,18,19,20,21,22,23]. Phenylpropanoid and flavonoid compounds are widely distributed in the plant defense system. When jujube trees were infected with phytoplasma, phenylpropanoid and flavonoid synthesis genes were up-regulated, suggesting that these compounds play a role in the defense response of jujube trees against phytoplasma [20]. Phytohormones can act as cellular signaling molecules that regulate plant immune responses to microbial pathogens, insect herbivores, and beneficial microorganisms [24]. The salicylic acid (SA) and jasmonic acid (JA) pathways were found to be antagonistic in phytoplasma-infected apples, which could be an effective mechanism for apples against phytoplasma invasion and provide a suitable defense response that leads to the establishment of recovery phenomena [25]. Jujube trees’ resistant to phytoplasma infection has a balance of high expression of JA and low expression of SA, a pattern that may be more beneficial for plant defense after phytoplasma infection [7]. Additionally, phytoplasma infection also alters the levels of growth hormones, such as gibberellins and abscisic acid, resulting in disease-related symptoms [26]. Zhou et al. [23] demonstrated that the JWB effectors SJP1 and SJP2 could activate the expression of growth hormone transporter proteins ZjPIN1c and ZjPIN3 by destabilizing ZjBRC1, thus inducing bushy symptoms in JWB plants. Chen et al. [27] demonstrated that the Zaofeng6 effector induced shoot proliferation by reducing the expression of ZjTCP7. However, the molecular response to JWB in asymptomatic jujube plants is not yet available.

In this study, we investigated the tolerance of Indian jujube cultivar ‘Cuimi’ (*Z. mauritiana* Lam.) to JWB by comparing its phenotype and phytoplasma concentrations with the susceptible cultivar ‘Huping’ (*Z. jujuba* Mill.). Furthermore, we conducted a comparative transcriptome analysis to elucidate the differences in defense mechanisms against phytoplasma invasion between the two cultivars.

## 2. Results

### 2.1. Indian Jujube ‘Cuimi’ Is High-Tolerant to JWB

**Figure 1:**
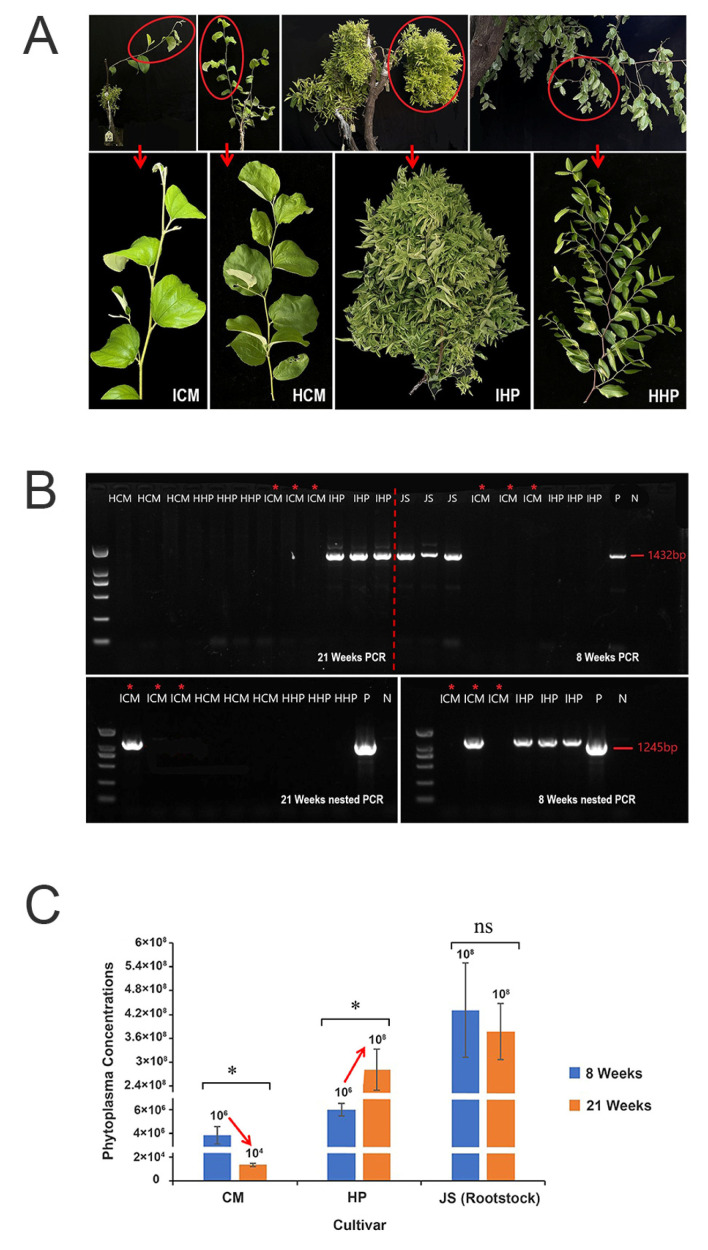
Phenotypes and results of phytoplasma-infected ‘Cuimi’ and ‘Huping’. (**A**) The phenotypes of grafted ‘Cuimi’ and ‘Huping’ at 21 WAG and two healthy controls. ICM: infected ‘Cuimi’; HCM: healthy ‘Cuimi’; IHP: infected ‘Huping’; HHP: healthy ‘Huping’. (**B**) Detection of phytoplasma in leaves of ‘Cuimi’ and ‘Huping’ at 8 and 21 WAG. P: positive; N: negative; JS: infected ‘Jinsixiaozao’ rootstocks. (**C**) Phytoplasma concentrations in two cultivars. * indicates significant differences at *p* < 0.05; ns indicates not significant.

To investigate the tolerance of ‘Cuimi’ cultivar to JWB, scions of healthy ‘Huping’ and ‘Cuimi’ were grafted onto JWB-infected ‘Jinsixiaozao’, and changes in growth phases were observed. As shown in Figure 1A, at 21 weeks after grafted (WAG), ‘Huping’ displayed severe symptoms of JWB, including yellowing, phyllody and witches’ broom, while ‘Cuimi’ grew normally and showed no discernible difference compared to its healthy control.

DNA samples were analyzed using nested PCR to detect the presence of phytoplasma. At 8 WAG, phytoplasma was not detected by directed PCR amplification from both cultivars grafted on diseased rootstocks (Figure 1B), however it was detected by nested PCR in all three samples of ‘Huping’ and one out of three samples of ‘Cuimi’. At 21 WAG, phytoplasma was detected by directed PCR amplification from ‘Huping’ but it was not detected from ‘Cuimi’ (Figure 1B). Furthermore, a phytoplasma band was only detected in one of the triplicate ‘Cuimi’ samples in the nested PCR (Figure 1B).

To measure the phytoplasma concentrations in two cultivars following grafting, quantitative PCR (qPCR) was used. At eight WAG, the phytoplasma concentrations were significantly higher in ‘Huping’ compared to ‘Cuimi’. Interestingly, as the grafting time increased, the phytoplasma concentrations significantly increased in ‘Huping’, while they significantly decreased in ‘Cuimi’ (Figure 1C). At 21 WAG, the phytoplasma concentrations remained significantly higher in ‘Huping’ compared to ‘Cuimi’. These findings suggested that the ‘Cuimi’ cultivar has a high tolerance to phytoplasma infection.

### 2.2. Screening for Differentially Expressed Genes in ‘Cuimi’ and ‘Huping’

The transcriptomes of two cultivars were analyzed to gain insight into their differential resistance against phytoplasma. After sequencing, a total of 237,252,603 clean reads were obtained, with all Q30 base percentages exceeding 91.85% (Table S1). Moreover, the percentages of clean reads mapped to the Chinese jujube reference genome ranged from 52.10% to 90.97% (Table S1).

**Figure 2:**
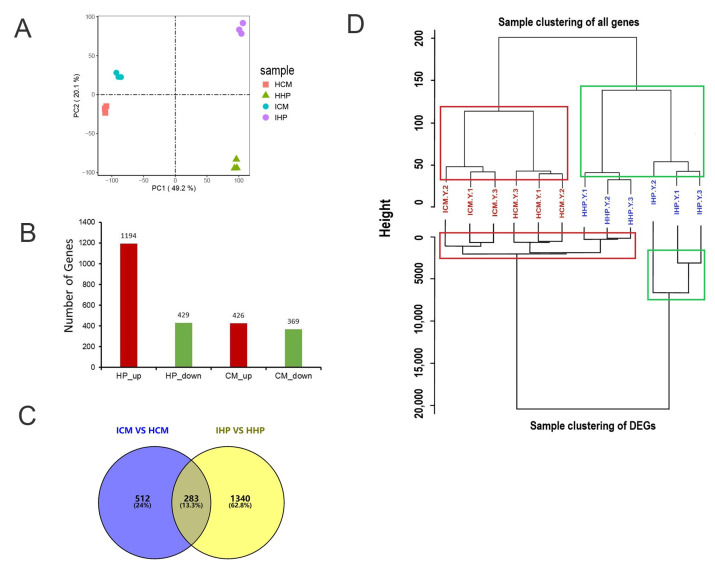
Screening for differentially expressed genes in ‘Cuimi’ and ‘Huping’. (**A**) Principal component analysis (PCA). (**B**) Number of DEGs in infected samples relative to healthy samples. Red indicates up-regulated DEGs; Green indicates down-regulated DEGs. (**C**) Venn diagram comparing DEGs in ‘Cuimi’ and ‘Huping’. (**D**) Sample clustering of all genes and DEGs in transcriptome analysis of ‘Cuimi’ and ‘Huping’. ICM: infected ‘Cuimi’; HCM: healthy ‘Cuimi’; IHP: infected ‘Huping’; HHP: healthy ‘Huping’.

Principal component analysis (PCA) results demonstrated the reliability of the data, with three replicates from each group being closely clustered together, thus ensuring the validity of subsequent analyses (Figure 2A). To investigate the differential resistance of two cultivars against phytoplasma infection, differentially expressed genes (DEGs) were analyzed. In JWB-infected ‘Huping’ compared to healthy ‘Huping’, 1194 DEGs were up-regulated, while 429 were down-regulated (Figure 2B). In JWB-infected ‘Cuimi’ compared to healthy ‘Cuimi’, 426 DEGs were up-regulated, and 369 were down-regulated (Figure 2B). In ‘Huping’, 1623 DEGs were identified, with 1340 unique DEGs (Figure 2C). In ‘Cuimi’, 795 DEGs were identified, including 512 unique DEGs (Figure 2C).

Based on the results of clustering analysis, it was observed that healthy ‘Huping’ clustered together with phytoplasma-infected ‘Huping’, and healthy ‘Cuimi’ clustered together with phytoplasma-infected ‘Cuimi’ (Figure 2D), when clustering was performed using all detected genes. However, when clustering was based on DEGs, it was found that phytoplasma-infected ‘Cuimi’ clustered with healthy ‘Cuimi’ and ‘Huping’, while phytoplasma-infected ‘Huping’ clustered separately (Figure 2D).

### 2.3. Gene Ontology (GO) Enrichment Analysis of DEGs

**Figure 3:**
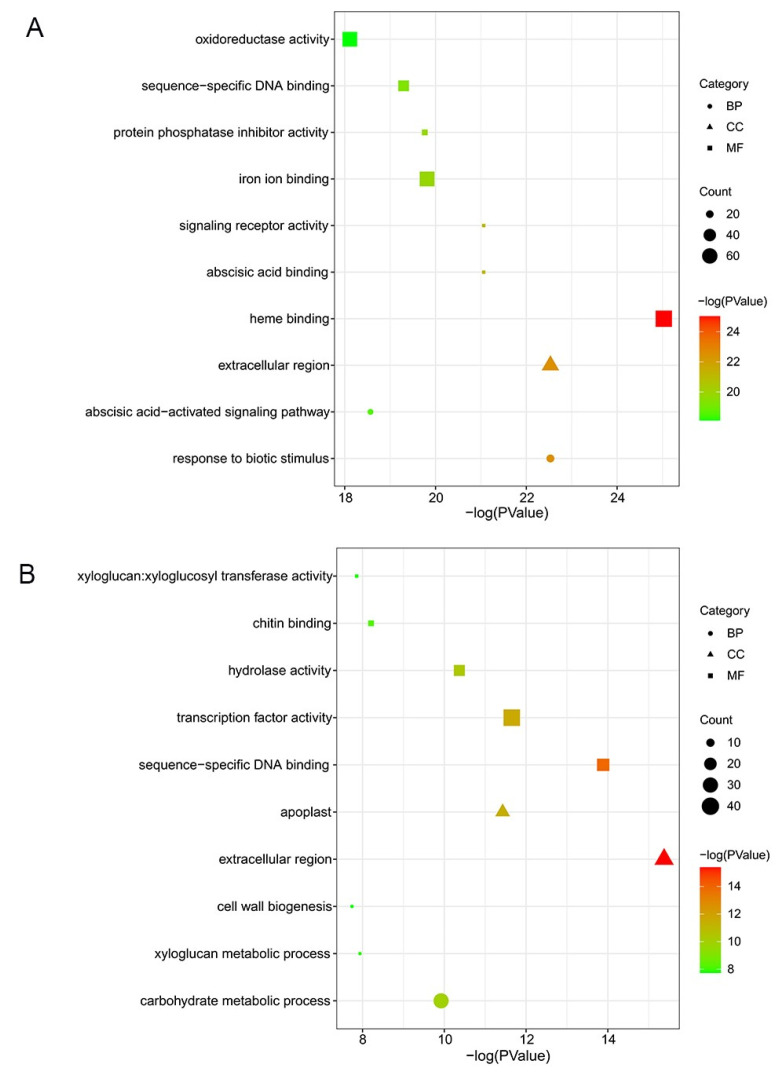
GO classification of DEGs in jujube cultivars. DEGs in ‘Huping’ (**A**) and in ‘Cuimi’ (**B**).

GO analysis showed the top 10 significant enrichment terms for each cultivar, including biological process (BP), molecular function (MF), and cell component (CC). In ‘Huping’, the top 3 enriched terms were heme binding, response to biotic stimulus, and extracellular region (Figure 3A), belonging to the MF, BP, and CC ontologies. The top 3 enriched terms were extracellular region, sequence-specific DNA binding, and transcription factor activity in ‘Cuimi’ (Figure 3B), belonging to the MF and CC ontologies. In the BP ontology, DEGs of ‘Cuimi’ were most significantly enriched in the carbohydrate metabolic process (Figure 3B).

**Figure 4:**
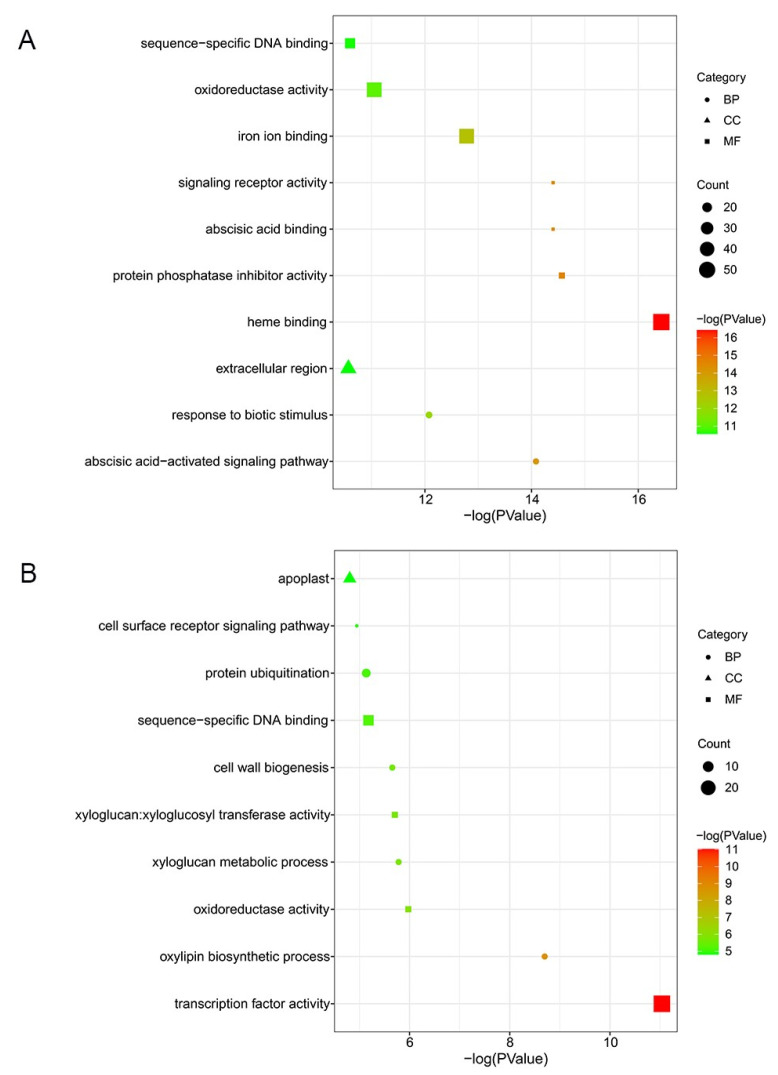
GO classification of unique DEGs in jujube cultivars. Unique DEGs in ‘Huping’ (**A**) and in ‘Cuimi’ (**B**).

To interpret the biological function of unique DEGs, we conducted GO annotation analysis. By contrasting DEGs detected in ‘Cuimi’ and ‘Huping’, we identified 1340 DEGs that were unique to ‘Huping’ (Figure 2B). Among these DEGs, the top 3 enriched terms were heme binding, protein phosphatase inhibitor activity, and abscisic acid binding in ‘Huping’, belonging to the MF ontology (Figure 4A). In the CC and BP ontologies, the most significantly enriched terms were extracellular region and the abscisic acid-activated signaling pathway, respectively(Figure 4A). We also identified 512 DEGs that were unique to ‘Cuimi’ (Figure 2B). Among these DEGs, the top 3 enriched terms were transcription factor activity, oxylipin biosynthetic process, and oxidoreductase activity in ‘Cuimi’ (Figure 4B), belonging to the MF and CC ontologies. In the CC ontology, the most significantly enriched term was the apoplast (Figure 4B).

### 2.4. Identification of Genes Associated with ‘Cuimi’ High Tolerance

**Figure 5:**
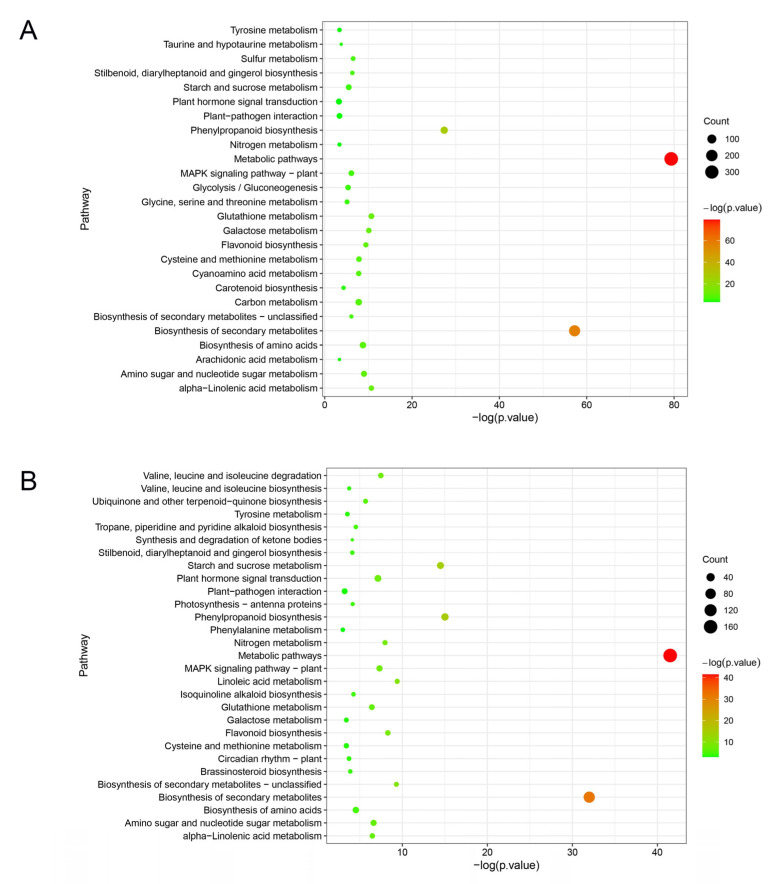
Pathways associated with DEGs in ‘Huping’ (**A**) and ‘Cuimi’ (**B**).

KEGG analysis revealed that DEGs of ‘Huping’ were significantly enriched in 26 pathways (Figure 5A), while DEGs of ‘Cuimi’ were significantly enriched in 29 pathways (Figure 5B). The two cultivars shared several common pathways, including metabolic pathways; biosynthesis of secondary metabolites; phenylpropanoid biosynthesis; glutathione metabolism; alpha-Linolenic acid metabolism; galactose metabolism; flavonoid biosynthesis; amino sugar and nucleotide sugar metabolism; biosynthesis of amino acids; cysteine and methionine metabolism; stilbenoid, diarylheptanoid and gingerol biosynthesis; MAPK signaling pathway; starch and sucrose metabolism; tyrosine metabolism; plant–pathogen interaction; nitrogen metabolism; and plant hormone signal transduction. Pathways exclusively enriched in ‘Cuimi’ included linoleic acid metabolism; valine, leucine and isoleucine degradation; ubiquinone and other terpenoid-quinone biosynthesis; tropane, piperidine and pyridine alkaloid biosynthesis; isoquinoline alkaloid biosynthesis; photosynthesis; synthesis and degradation of ketone bodies; brassinosteroid biosynthesis; valine, leucine and isoleucine biosynthesis; circadian rhythm—plant; and phenylalanine metabolism. Meanwhile, pathways exclusively enriched in ‘Huping’ were cyanoamino acid metabolism; carbon metabolism; sulfur metabolism; glycolysis/gluconeogenesis; glycine, serine and threonine metabolism; taurine and hypotaurine metabolism; and arachidonic acid metabolism. The most significantly enriched pathway in both cultivars was metabolic pathways.

Expression analysis of genes was conducted for the common pathways between the two cultivars. In the plant hormone signal transduction and biosynthesis pathways, ‘Huping’ displayed down-regulated genes related to auxin (IAA), and up-regulated genes related to JA and SA, while ‘Cuimi’ showed up-regulated genes related to IAA and SA, and down-regulated genes related to JA (Table S2). In the galactose metabolism and amino sugar and nucleotide sugar metabolism pathways, genes that showed significant expression differences between the two cultivars included 6-phosphofructokinase 1, inositol 3-alpha-galactosyltransferase, stachyose synthetase, and chitinase (Table S2). In secondary metabolism-related pathways, genes with significant expression differences between the two cultivars were cinnamoyl-CoA reductase, feruloyl CoA ortho-hydroxylase and peroxidase (POD) (Table S2).

**Figure 6:**
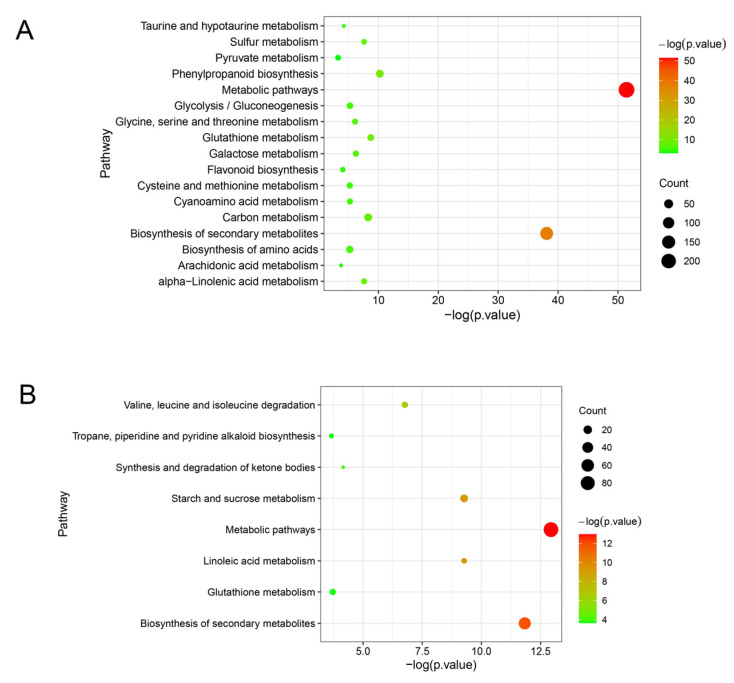
Pathway enrichment results for DEGs unique to ‘Huping’ (**A**) and ‘Cuimi’ (**B**).

The unique DEGs in ‘Huping’ were significantly enriched in 17 pathways (Figure 6A), while the unique DEGs in ‘Cuimi’ were significantly enriched in 8 pathways (Figure 6B). Three pathways in ‘Cuimi’ were same to ‘Huping’, i.e., metabolic pathways, biosynthesis of secondary metabolites and glutathione metabolism. Fourteen pathways were unique to ‘Huping’, including phenylpropanoid biosynthesis; carbon metabolism; alpha-Linolenic acid metabolism; sulfur metabolism; galactose metabolism; glycine, serine and threonine metabolism; glycolysis/gluconeogenesis; cyanoamino acid metabolism; biosynthesis of amino acids; cysteine and methionine metabolism; taurine and hypotaurine metabolism; flavonoid biosynthesis; arachidonic acid metabolism; and pyruvate metabolism. Five pathways were unique to ‘Cuimi’, including linoleic acid metabolism; starch and sucrose metabolism; valine, leucine and isoleucine degradation; synthesis and degradation of ketone bodies; and tropane, piperidine and pyridine alkaloid biosynthesis. Among the pathways enriched with unique genes in both cultivars, only the starch and sucrose metabolism pathway in ‘Cuimi’ was analyzed. In this pathway, most of the genes were down-regulated in ‘Cuimi’ compared to ‘Huping’, such as beta-amylase and sucrose-phosphate synthase genes (Table S2).

We further analyzed 226 genes with significant differences in gene expression between the two cultivars. These genes were significantly enriched in nine pathways: metabolic pathways, plant hormone signal transduction, galactose metabolism, MAPK signaling pathway, nitrogen metabolism, alpha-Linolenic acid metabolism, linoleic acid metabolism, biosynthesis of secondary metabolites, and plant–pathogen interaction (Table S3). We categorized these genes into three groups based on their expression patterns in the two cultivars: high/low-low/high, high/low-no significant expression changes, and high/low-high/low (Table S4). The genes in the high/low-low/high and high/low-no significant groups exhibited opposite expression patterns in the two cultivars. Consequently, we designated 194 genes from these two groups as key genes associated with JWB high tolerance.

In the MAPK signaling pathway (Table S5), five genes were up-regulated in ‘Cuimi’, which encode respiratory burst oxidase (RBOH), EIN3-binding F-box protein 1 (EBF1), protein phosphatase 2C (PP2C), mitogen-activated protein kinase 12 (MAPK 12), and basic endochitinase B; while one gene encoding 1-aminocyclopropane-1-carboxylate synthase 1/2/6 was down-regulated. In ‘Huping’, three genes encoding RBOH, PP2C, and MAPK 12 were down-regulated, while two genes were up-regulated, which encode 1-aminocyclopropane-1-carboxylate synthase 1/2/6 and basic endochitinase B. In the plant–pathogen interaction pathway (Table S5), two genes encoding enhanced disease susceptibility 1 protein (EDS1) and RBOH were up-regulated in ‘Cuimi’, while calcium-binding protein CML genes were down-regulated. Conversely, two genes encoding EDS1 and RBOH were down-regulated in ‘Huping’, and CML genes were up-regulated.

## 3. Discussion

### 3.1. The High Tolerance to JWB of ‘Cuimi’

Previous studies have identified JWB-resistant cultivars, such as ‘Xingguang’ and ‘T13’ [6,7]. These resistant cultivars only showed symptoms slightly at the initial stage after grafting inoculation, while eventually reversing to normal growth. However, in this study, phytoplasma-infected ‘Huping’ displayed severe symptoms of yellowing, phyllody, and witches’ broom, while phytoplasma-infected ‘Cuimi’ grew normally without any apparent visible symptoms of JWB. The phytoplasma concentration increased with grafting time for ‘Huping’, while it decreased with grafting time for ‘Cuimi’. These findings indicated that ‘Cuimi’ has a certain mechanism to inhibit the growth and/or reproduction of JWB phytoplasma in infected tissues. In addition, we also investigated the tolerance of 5 other Indian jujube cultivars, including ‘50’, ‘Niunai’, ‘Gaolang No.1’, ‘Misi’, and ‘Pingguo’, which similarly showed high tolerance (Pang et al., unpublished data). Phytoplasma-infected young Ber in India can cause symptoms such as phyllody, yellowing and proliferation (growth of shoots from floral organs) [13], but no JWB infection in *Z. mauritiana* Lam. has been reported in China. The differential phenotype of *Z. mauritiana* after infection with phytoplasma in China and India may be due to different growth environments. The impact of environmental changes on the severity of plant diseases is significant. Environmental factors have an influence on plant resistance pathways and defense hormone networks, while temperature and humidity affect pathogen virulence mechanisms, reproduction, and survival [28]. Additionally, it may be also due to different phytoplasma strains infecting the plants, as plant pathogens have host specificity, and plants vary in their sensitivity to different phytoplasma strains [29].

### 3.2. Response of JWB Susceptible Jujube Cultivar to Phytoplasma Infection

Ye et al. [30] and Wang et al. [20] reported that phytoplasma infection markedly inhibits photosynthesis and the biosynthesis of chlorophyll and peroxisomes but increases carbohydrate metabolism and the biosynthesis of secondary metabolites. Additionally, during jujube infection, genes involved in plant–pathogen interaction were initially down-regulated and subsequently up-regulated. Our transcriptome data of ‘Huping’ showed similar patterns, consistent with existing research on carbohydrate metabolism and secondary metabolite biosynthesis. A common effect of phytoplasma infection is the accumulation of glucose, fructose, sucrose, and starch in infected plants, such as periwinkle, tobacco, papaya, coconut, and maize [31,32,33,34]. Phytoplasmas may rely on fructose or glucose as an energy source, since they do not possess enzymes for sucrose utilization [35]. Phenylpropanoid and flavonoid compounds are widely distributed in the plant defense system [36]. In our study, the genes involved in the plant–pathogen interaction of ‘Huping’ were mostly up-regulated, consistent with the late-stage (39 WAG) data of jujube infection [20]. This suggests that phytoplasmas completely colonized the host tissues in the JWB-susceptible cultivar, resulting in a significant upregulation of genes involved in plant–pathogen interactions. Previous studies have indicated that in jujube trees infected with phytoplasma, most of the DEGs involved in JA and SA biosynthesis and signal pathways were up-regulated, such as lipoxygenase, 4-coumarate-CoA ligase, and phenylalanine ammonia-lyase, while some DEGs related to IAA were down-regulated, which is similar to our findings on ‘Huping’ [20,30]. These results indicated general conclusions for jujube after phytoplasma infection, including upregulation of genes related to carbohydrates and secondary metabolites and changes in the expression of genes related to plant hormones. The consistency of DEGs and expression patterns with previous studies on the susceptible cultivar ‘Huping’ indicated the reliability of our transcriptome data.

### 3.3. Response of JWB Tolerant Jujube Cultivar to Phytoplasma Infection

After analyzing Go results, some unique terms were identified in ‘Cuimi’ including transcription factor activity, xyloglucan metabolic process, xyloglucan: xyloglucosyl transferase activity, cell wall biogenesis, protein ubiquitination, cell surface receptor signaling pathway, carbohydrate metabolic process, oxylipin biosynthetic process, hydrolase activity, chitin binding, and apoplast. These terms may relate to the normal development and growth of ‘Cuimi’ under phytoplasma infection. Key genes related to the high tolerance of JWB were also identified, which included some genes related to ROS, Ca^2+^ sensors, protein kinases, transcription factors (TFs), lignin, and hormones (Table S6). Previous studies have reported that genes related to ROS, protein kinases, TFs, and hormones are also found in JWB-resistant cultivars. Liu et al. [17] believed that protein kinases and TFs such as MAPK, ERF, and zinc finger proteins are related to the resistance of ‘Xingguang’. In the recovery process of jujube trees infected with phytoplasma treated with tetracycline, it was found that genes related to JA were down-regulated [37]. Xue et al. [38] compared the expression of WRKY genes between resistant and susceptible cultivars and found that the expression of some WRKY genes was lower in resistant cultivars than in susceptible ones, which may play a negative regulatory role in the pathogen tolerance of plants. Wang et al. [7] believed that key resistance genes primarily participate in the signal transduction of hormones and ROS. In the resistance model, the generation of ROS can accumulate JA, which then antagonizes the SA content, while MAPKK6 and MYC2 also play a negative regulatory role in resistance. An increased zeatin-to-auxin ratio is a potential mechanism for enhancing resistance to phytoplasma infection [7]. In our study, the gene expression of POD was significantly up-regulated in ‘Cuimi’ during the infection of phytoplasma, which may be a defensive response. The increased expression of RBOH in a high tolerance cultivar may mediate the immune response. Numerous protein kinases, such as MAPKs, calcium-dependent protein kinases, and receptor-like kinases (RLKs), are essential for immune signaling [39]. Serine/threonine protein kinases (STKs) are crucial for plant defense responses as they play a significant role in detecting and transmitting signals from pathogens [40]. Lin et al. [41] found that AvrPphB SUSCEPTIBLE1-LIKE13 (PBL13) kinase negatively regulates plant innate immunity to pathogenic bacteria and can associate with RBOH before pathogen perception. The kinases in key genes, such as MAPK and STK, may serve as important signaling molecules in the high tolerance of ‘Cuimi’. Numerous transcription factors are crucial for both innate resistance and the specific recognition of pathogen effectors. Multiple transcription factors in key genes showed differential expression between the two cultivars and were down-regulated in ‘Cuimi’, playing an important role in the high tolerance to JWB. In addition, genes related to JA signaling transduction and biosynthesis, such as jasmonate ZIM domain-containing protein (JAZ) and lipoxygenase (LOX), were down-regulated in infected ‘Cuimi’, indicating that the decrease in JA content contributes to the high tolerance of ‘Cuimi’ to JWB (Table S2). Some key genes were the same as those related to JWB-resistant cultivars reported earlier. These key genes play an important role in the high tolerance mechanism of Indian jujube ‘Cuimi’ to phytoplasma.

Furthermore, the key genes associated with high tolerance to JWB that are related to Ca^2+^ sensors and lignin have not been reported. Ca^2+^ binding proteins, such as Calmodulin (CAM) and CML, act as Ca^2+^ sensors and relay Ca^2+^ signals into down-stream signaling events [42]. Ca^2+^ signaling in plant cells is an essential and early event during plant–microbe interactions [43]. Silencing the expression of APR134, a CML gene in tomatoes, suppresses the hypersensitive response (HR), whereas overexpression of CML 43, an ortholog of APR134 in Arabidopsis, accelerates HR [44]. In the infected ‘Cuimi’, the CML gene was significantly down-regulated, indicating that it might be a negative regulatory factor related to the high tolerance of JWB. The deposition of lignin can form a physical obstruction that restricts the entry and propagation of pathogens [45]. The function of Rice Snl6, a member of the cinnamoyl-CoA reductase-like gene family, is essential for the activation of NPR1 homolog 1 (NH1)-mediated defense response against *Xanthomonas oryzae pv. oryzae* [46]. In infected ‘Cuimi’, the cinnamoyl-CoA reductase-like SNL6 gene was significantly up-regulated, suggesting that it may cause lignin deposition, thereby limiting the growth of phytoplasma and also mediating ‘Cuimi’s response to the pathogen. In the future, we will explore and validate the molecular mechanisms of these key genes in high tolerance Indian jujube cultivar.

## 4. Materials and Methods

### 4.1. Plant Materials

‘Cuimi’ (*Z. mauritiana* Lam.) and ‘Huping’ (*Z. jujuba* Mill.) scions were grafted onto the diseased ‘Jinsixiaozao’ (*Z. jujuba* Mill.) cultivar’ rootstocks at the National Key Base for Improved Chinese Jujube Cultivar (Cangzhou, China). Phenotypic observations were performed at 21 weeks after grafting. Molecular tests and phytoplasma concentrations were conducted at 8 and 21 weeks after grafting, respectively.

### 4.2. Detection of JWB Phytoplasma Using Nested PCR

DNA was extracted from collected samples during both periods using the Plant DNA Kit (Vazyme, China) according to the manufacturer’s instructions. Nested PCR was performed to detect the presence of phytoplasma as described previously [47]. Two rounds of nested PCR were implemented with two sets of primers and temperature cycles, as shown in Tables S7 and S8. For the nested PCR reaction, a 50-fold diluted template generated by R16mF2/R2 primers was used. Then, the nested PCR products were detected by 1% agarose gel electrophoresis, and observed and photographed using a gel imaging system for storage.

### 4.3. Quantitative PCR (qPCR)

The DNA samples were amplified using phytoplasma-specific primers R16mF2/R2 (Table S7) to obtain a 1245 bp fragment of JWB phytoplasma. This fragment was then cloned into the pMD18-T vector (Takara Bio, Kyoto, Japan) and subsequently sequenced to confirm the successful integration of the target fragment. The molecular weight of the recombinant plasmid was calculated using DNAMAN software, and the plasmid concentration was measured using Nanodrop8000 (Thermo Fisher Scientific, Waltham, MA, USA). Plasmid copy number formula: DNA concentration/DNA molar mass × 6.02 × 10^23^. The number of plasmid copies per microliter was calculated according to this formula. Finally, a standard curve was established by performing real-time fluorescence quantitative PCR on plasmid gradient dilutions with JWB16S primers (Table S7).

Phytoplasma concentrations were quantified using quantitative fluorescence PCR. The resulting phytoplasma content was divided by the weight of the tissue at the time of DNA extraction and then divided by 2 (16S rDNA in phytoplasma is a double copy) to obtain the phytoplasma content per unit of fresh tissue weight [48].

### 4.4. RNA-Seq Library Construction and Sequencing

Total RNA was extracted from infected and healthy leaves of two cultivars, each with three biological replicates, after 21 WAG using an RNAprep pure Plant Kit (TIANGEN, Beijing, China) according to the manufacturer’s instructions. Subsequently, the quality and integrity of the total RNA were evaluated using the Qubit@ 2.0 Fluorometer (Thermo Fisher Scientific, Waltham, MA, USA) and the Bioanalyzer 2100 system (Agilent Technologies, Santa Crala, CA, USA) in the company Novogene (Beijing, China). High-quality RNA samples were employed for the generation of cDNA libraries. To elaborate, mRNA was isolated using oligo (dT) magnetic beads, and then fragmented randomly with fragmentation buffer. Using these short fragments, the double-stranded cDNA was synthesized with random hexamer primers and DNA polymerase I, followed by an RNase H treatment. The cDNA was purified, end-paired, and ligated with adaptors after “A” bases were added. It was then fragmented into approximately 200 bp pieces with AMPure XP beads for PCR applications. The Illumina HiSeq 2500 system from Novogene (Beijing, China) was used to perform paired-end sequencing on the twelve libraries.

### 4.5. Data Analysis

The raw data were filtered with trim_galore v.0.4.1 to obtain clean data, and quality control was performed with fastqc v.0.11.9. The reference genome and gene annotation files were based on Ziziphus jujuba (assembly ZizJuj_1.1) available on NCBI. All clean data were compared with reference genes using hisat2 v.2.2.1 and quantified using featureCounts v.2.0.1. Differential expressed genes were carried out using DESeq2 v.1.30.1, with the screening criteria |Fold Change| > 1.5 and Q-value < 0.05. The gene expression values were normalized using FPKM (fragments per kilobase of exon model per million mapped fragments) normalization. Key analysis was to identify genes with significant differences in gene expression among the differential genes of two cultivars.

DEGs were subjected to gene ontology (GO) and Kyoto Encyclopedia of Genes and Genomes (KEGG) enrichment analyses by DAVID (https://david.ncifcrf.gov/, accessed on 1 July 2022) (*p*-value < 0.01) and KOBAS software (http://kobas.cbi.pku.edu.cn/home.do, accessed on 5 July 2022) (*p*-value < 0.05).

### 4.6. Statistical Analysis

All statistical analyses were performed using R software v. 3.6.1. T-test was performed to test the significance level (* *p* < 0.05, ** *p* < 0.01, and *** *p* < 0.001). The values were expressed as the means of three biological replicates. All samples were normally distributed with homogeneity of variance. Three independent replicates of measurements were performed for each sample. All chart data were shown as mean ± error bars’ deviation.

## 5. Conclusions

In summary, we confirmed that Indian jujube ‘Cuimi’ is high-tolerant to JWB based on phenotype and molecular evidence. We designated 194 genes as key genes related to JWB high tolerance. The gene expression of ROS, Ca^2+^ sensors, TFs, protein kinases, lignin, and hormones plays an important role in the JWB high tolerance mechanism of ‘Cuimi’. Furthermore, we observed that CML genes were significantly down-regulated in infected ‘Cuimi’, suggesting that the CML gene serves as a negative regulatory factor related to the high tolerance of JWB. The cinnamoyl-CoA reductase-like SNL6 gene was significantly up-regulated in infected ‘Cuimi’, leading to lignin deposition and limiting the growth of phytoplasma while mediating ‘Cuimi’s immune response to phytoplasma.

## Data Availability

All needed genome sequences and genome annotation files of jujube were obtained from the NCBI database (https://www.ncbi.nlm.nih.gov/assembly/GCF_000826755.1/, accessed on 6 February 2015). The transcriptome sequencing data of different samples in this study were retrieved from the GSA database (https://ngdc.cncb.ac.cn/gsa/, accessed on 28 March 2023) and these RNA-sequencing reads were previously deposited with GSA under accession code PRJCA015907. In addition, all databases are included in this published article and its Supplementary Materials.

## Supplementary Materials

The following supporting information can be downloaded at: https://www.mdpi.com/article/10.3390/plants12112082/s1, Table S1: The statistical analysis of clean data by transcriptome sequencing and mapped results; Table S2: Gene expression changes in KEGG pathways of ‘Cuimi’ and ‘Huping’; Table S3: Pathway enrichment analysis of 226 genes; Table S4: Expression patterns of 226 genes; Table S5: Changes in gene expression of KEGG pathways; Table S6: Expression of some key genes; Table S7: Primers used in this study; Table S8: Thermal cycles of nested PCR.
